# Prediction of aortic dilation in Turner syndrome - enhancing the use of serial cardiovascular magnetic resonance

**DOI:** 10.1186/1532-429X-15-47

**Published:** 2013-06-06

**Authors:** Kristian H Mortensen, Mogens Erlandsen, Niels H Andersen, Claus H Gravholt

**Affiliations:** 1 Department of Endocrinology and Internal Medicine (MEA), Aarhus University Hospital, Aarhus, Denmark; 2 Department of Radiology, Addenbrooke’s Hospital, Cambridge University Hospitals, Cambridge, United Kingdom; 3 Section of Biostatistics, Department of Public Health, Aarhus University, Aarhus, Denmark; 4 Department of Cardiology, Aarhus University Hospital, Aarhus, Denmark

**Keywords:** Aorta, Follow-up studies, Congenital heart disease, Hypertension, Cardiovascular magnetic resonance, Outcomes research, Turner syndrome, Bicuspid aortic valve

## Abstract

**Background:**

Identification of the subset females with Turner syndrome who face especially high risk of aortic dissection is difficult, and more optimal risk assessment is pivotal in order to improve outcomes. This study aimed to provide comprehensive, dynamic mathematical models of aortic disease in Turner syndrome by use of cardiovascular magnetic resonance (CMR).

**Methods:**

A prospective framework of long-term aortic follow-up was used, which comprised diameters of the thoracic aorta prospectively assessed at nine positions by CMR at the three points in time (baseline [n = 102, age 38 ± 11 years], follow-up [after 2.4 ± 0.4 years, n = 80] and end-of-study [after 4.8 ± 0.5 years, n = 78]). Mathematical models were created that cohesively integrated all measurements at all positions, from all visits and for all participants, and using these models cohesive risk factor analyses were conducted based on which predictive modeling was performed on which predictive modelling was performed.

**Results:**

The cohesive models showed that the variables with effect on aortic diameter were aortic coarctation (P < 0.0001), bicuspid aortic valves (P < 0.0001), age (P < 0.0001), diastolic blood pressure (P = 0.0008), body surface area (P = 0.015) and antihypertensive treatment (P = 0.005). Oestrogen replacement therapy had an effect of borderline significance (P = 0.08). From these data, mathematical models were created that enabled preemption of aortic dilation from CMR derived aortic diameters in scenarios both with and without known risk factors. The fit of the models to the actual data was good.

**Conclusion:**

The presented cohesive model for prediction of aortic diameter in Turner syndrome could help identifying females with rapid growth of aortic diameter, and may enhance clinical decision-making based on serial CMR.

## Background

A 100-fold increased risk of aortic dissection and rupture in Turner syndrome (TS) calls for sensitive and specific risk factors in order to appropriately triage to follow-up and interventions [1,2]. Aortic dilation is the principal risk factor for aortic events, [3] which in TS affects both the ascending and descending thoracic aorta [4]. Hypertension, bicuspid aortic valve, aortic coarctation and 45,X karyotype are all factors that increase the likelihood of aortic dilation, accelerated aortic growth and aortic dilation [5-8]. These risk factors, however, fail to predict aortic dissection and rupture in a large proportion of females with TS and current clinical triage must be optimised [4].

The risk of aortic events is presently estimated from static measurements of aortic diameter, [9] assessing diameters at individual aortic positions and placing them into a general comparison with normal calibre at this position [5-8]. This approach fails to appreciate the thoracic aorta as a complex structure because it does not include the dynamic interrelation between different measurement positions. There is also a risk of oversimplifying risk assessment, since the same aortic diameter will not confer the same risk in females with or without risk factors present such as 45,X karyotype, hypertension, age or enlarged aortic diameter at other positions. In contemporary best practice only body size is controlled for [5]. Furthermore, from a mathematical viewpoint the single-site approach runs the risk of both failing to acknowledge and overemphasising the relevance of risk factors. These aspects together with a shortage on longitudinal insight in TS are likely to contribute to our failure to accurately predict the risk of aortic dissection in this cohort and enhanced insight is pivotal before outcomes can be improved [4].

The present study aimed to provide comprehensive dynamic mathematical models, of cross-sectional and longitudinal nature, for aortic disease in TS that integrated multiple measuring positions as well as known risk factors for aortic disease. The aim was to enable more valid prediction of aortic disease in TS and to optimise clinical use of cardiovascular magnetic resonance (CMR) studies.

## Methods

### Study population

Females with karyotypically proven TS (*n* = 102) were recruited through the Danish National Society of Turner Syndrome Contact Group and a tertiary endocrine outpatient clinic. Exclusion criteria were malignancy, liver disease and contraindications to CMR (including mechanical aortic valve prostheses). The participants were examined at baseline, follow-up and end of study using CMR. As previously described, echocardiography and 24-hour ambulatory blood pressures were also performed [6,10,11].

### Cardiovascular magnetic resonance

CMR was performed with a 1.5 T whole-body scanner (ACS-NT, Philips Medical Systems; maximum gradient performance 30 Tesla per meter amplitude, slew rate 150 T/m/sec). A 5-element cardiac coil was used. After initial scouts, a three-dimensional data stack (27 cm (anterior-posterior) × 15 cm × 36 cm (left-right)) covering the entire thoracic aorta was acquired. A contrast-free, nearly isotropic, fat-saturated, three-dimensional steady-state free precession and electrocardiogram-triggered gradient echo sequence (250 ms diastolic acquisition window) with a respiratory navigator was used [12]. All studies were performed by the same staff and in the same scanner.

Two experienced observers performed a systematic analysis of the aortic data sets for maximum aortic diameters with dedicated software (Systematic Software Engineering, Aarhus, Denmark). The post-processing methodology allowed reconstruction of the 3D stack of data in any plane with measurements of aortic diameters that were truly perpendicular to the aortic wall and the aortic axis at the measurement position, as described elsewhere [11]. The observers were blinded to the clinical data of the patient. Aortic or extra-aortic landmarks guided the measurement positions. The positions were: (i) aortic sinuses (measuring cusp-to-opposing-cusp diameter at the point of the maximum aortic diameter in the aortic sinus); (ii) ascending aorta at the sinotubular junction; (iii) mid-ascending aorta at the level of the inferior margin of right pulmonary artery; (iv) distal ascending aorta immediately proximal to brachiocephalic artery; (v) proximal aortic arch between the brachiocephalic and left carotid artery arteries; (vi) distal aortic arch immediately proximal to left subclavian artery; (vii) aortic isthmus immediately distal to the left subclavian artery; (viii) proximal descending aorta between the left pulmonary artery and the top of left atrium; and (ix) distal descending aorta at the most caudal border of the left atrium. No systematic bias was seen on Bland-Altman variability analyses of measurement error, when assessed for inter-observer and intra-observer variability [11]. Intra-observer variability was for the above measurement positions: (i) -0.04 (−1.9;1.8) mm; (ii) 0.02 (−1.8;1.9) mm; (iii) -0.1 (−1.9;1.8) mm; (iv) -0.1 (−1.9;2.1) mm; (v) 0.20 (−1.6;2.0) mm; (vi) 0.01 (−1.7;1.7) mm; (vii) 0.1 (−1.6;1.4) mm; (viii) 0.08 (−1.5;1.4) mm; and (ix) -0.06 (−1.6;1.7) mm [11]. Inter-observer variability was: (i) -0.1 (−2.1;2.3) mm; (ii) -0.3 (−2.3;1.8) mm; (iii) -0.1 (−1.9;1.4) mm; (iv) 0.1 (−1.6;1.7) mm; (v) -0.2 (−1.4;1.9) mm; (vi) -0.01 (−1.6;1.4) mm; (vii) -0.1 (−1.4;1.9) mm; (viii) 0.08 (−1.1;1.9) mm; and (ix) 0.1 (−1.2;1.5) mm [11]. Morphological aortic abnormalities were detected according to previously described principles [10].

### Ethics

Informed consent was obtained from each participant, and the study protocol conformed to the ethical guidelines of the 1975 Declaration of Helsinki as reflected in a priori approval by Aarhus County Ethical Scientific Committee (Denmark) (# 20010248).

### Statistical methods

#### Descriptive statistics

Mathematical computations were performed using SPSS 18.0. Normal distribution of data was tested with Shapiro-Wilk test, and when not normally distributed the data was transformed by use of natural logarithmic conversion. Continuous variables are expressed as means ± standard deviations with ranges. Data was compared using paired *t*-test (after testing for equal variances by Levene’s test). P < 0.05 was considered significant.

#### Mathematical models

The spatial relationship between the nine aorta measurement positions and the temporal dynamics of the three time points were analysed with a mixed model on the natural logarithm of aortic diameter. The risk factors in the fixed part of the model were: age, diastolic blood pressure, aortic valve morphology, aortic coarctation, antihypertensive medicines and body surface area at each time point. These variables were entered in the model with position dependent coefficients. The random parts of the model were composed of 3 terms: (i) an unstructured covariance matrix describing the spatial relationship between the positions, (ii) a first order auto-regression describing the temporal dynamics, and (iii) position dependent measurement errors, the first two terms were put together in a so-called Kronecker product (see Additional file 1 for further details) [13]. The model was fitted with SAS/STAT 9.3 PROC MIXED [14]. The model can be used to make individual baseline predictions using information on individual risk factors even without any prior information about previous measured aorta diameter. The model could also be rewritten as a Kalman filter [15] due to the autoregressive term, in order to forecast aortic diameter from expected developments in the risk factors and previously obtained aorta measurements. The forecasting model was programmed with the Kalman filter commands in SAS/IML 9.3 [16].

The chosen mathematical model could in a simultaneous way handle both the spatial and temporal relationships in the data, and thus took full advantage of the design with repeated (over time) measurements for multiple positions. Similar mathematical techniques have been successfully applied in real time medical imaging, e.g. in electrocardiography and computerised tomography (please see Zhang et al. [17] for further references).

The model forecasting aortic diameter is available for clinicians and researchers at http://www.biostat.au.dk/MERL/Aorta_Prediction_model.htm.

## Results

### Study population

The baseline cohort consisted of 102 females with TS [6]. Of these, 78 females (age 38 ± 9.9 (19 – 62) years) with TS (61% 45,X, and 39% other karyotypes) were followed for 4.8 ± 0.5 (3.5-5.7) years, and they were examined thrice. The loss to follow-up over the entire study was due to: (i) exclusion due to chronic Stanford type A aortic dissection (n = 1), aortic valve surgery (n = 1) and mitral valve surgery (n = 1); (ii) deaths (sudden death of unknown cause (n = 2), complications of elective aortic aneurysm surgery (n = 1), and metastatic gastric adenocarcinoma (n = 1)), (iii) withdrawal of consent for non-health related reasons (n = 13), and (iv) technically suboptimal CMR studies where repeat imaging was declined (n = 4). The cohorts that participated at each visit (baseline, follow-up and end of study) are described in Table 1. Data for baseline and follow-up have been described previously [6,11].

**Table 1:** Females with Turner syndrome followed with three visits over 4.8 years using CMR

		**Baseline**	**Follow up**	**End of study**
**Participants** (n)		102	89	82
**Total exit from study** (n)		-	13	20
**Technically successful study** (n)		102 (100%)	80 (90%)	78 (95%)
**Age** (years)		38 ± 11	41 ± 11	43.0 ± 10
**Follow up time** (years)		-	2.4 ± 0.4	4.8 ± 0.5
**Body surface area** (m^2^)		1.5 ± 0.2	1.5 ± 0.2	1.5 ± 0.1
**Karyotype** (45,X/other)		58%/42%	60%/40%	61%/39%
**Oestrogen replacement therapy**		86%	85%	82%
**Antihypertensive treatment**		29%	28%	55%
**Statin treatment**		6%	9%	13%
**Diabetes**		5%	8%	12%
**Previous growth hormone treatment**		27%	26%	22%
**Ambulatory blood pressure**				
24-hour systolic (mm Hg)		122 ± 14	122 ± 14	117 ± 13
24-hour diastolic (mm Hg)		77 ± 11	78 ± 11	75 ± 8
24-hour heart rate (beats/min)		76 ± 10	77 ± 9	74 ± 9
**Aortic dimensions** (mm)				
Aortic sinus		29.1 ± 4.0	29.2 ± 3.0 *	31.0 ± 4.4 *
Sinotubular junction		25.4 ± 4.7	25.3 ± 4.3	26.3 ± 4.6 *
Mid-ascending aorta		27.4 ± 6.7	27.5 ± 5.0	28.6 ± 5.2 *
Distal ascending aorta		25.4 ± 4.0	25.5 ± 3.6	25.8 ± 3.7
Proximal aortic arch		23.5 ± 3.7	23.4 ± 3.6	23.9 ± 3.5
Mid aortic arch		20.5 ± 2.6	20.5 ± 2.7	20.7 ± 2.6
Distal transverse aortic arch		19.3 ± 2.4	19.3 ± 2.3	19.4 ± 2.3
Proximal descending		19.4 ± 2.9	19.5 ± 2.8	19.8 ± 3.4
Distal descending aorta		18.2 ± 2.4	18.2 ± 2.2	18.3 ± 2.5
**Aortic valve and aortic anomalies**				
Bicuspid aortic valve		25%	28%	30%
Aortic stenosis †		12% (7%/4%/1%)	11% (8%/3%/-)	13% (9%/4%/-)
Aortic regurgitation †		22% (16%/5%/1%)	21% (18%/3%/-)	23% (17%/5%/1%)
Elongated transverse aortic arch		47%	48%	48%
Aortic coarctation ‡		12%	11%	10%

Continuous variable are expressed as means ± standard deviations.

* P < 0.05 using Student’s paired *t*-test to compare baseline and end of study.

† Percentages describe grades of dysfunction (mild/moderate/severe). There was no significant change in valvar indices during the follow-up.

‡Morphological aortic coarcation on CMR.

### Aortic diameter

**Figure 1:**
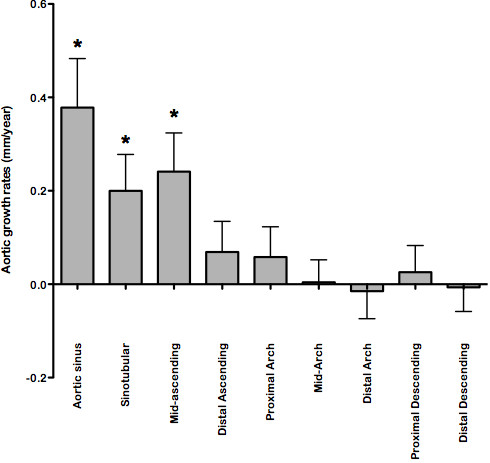
**Aortic growth rates (mean and 95% confidence intervals) during 4.8 ± 0.5 years of CMR in Turner syndrome (n = 78).** * P < 0.05 when comparing baseline to follow-up using Students independent *t*-test.

During follow-up aortic diameter increased significantly at sinus level, sinotubular junction and mid-ascending aorta (P < 0.05) (Table 1) with a trend for growth also in the distal ascending aorta and the proximal aortic arch (P < 0.07). The remaining aortic positions were unchanged. Aortic growth rates ranged from 0.20 ± 0.34 to 0.38 ± 0.46 mm/year for the three most proximal measurements in the ascending aorta (Figure 1).

### Mathematical modelling

In a mathematical model using all available data points we first examined whether the different variables had any effect on aortic diameter at the different positions along the thoracic aorta. Of these variables (Table 2), the significant ones were aortic coarctation (P < 0.0001), bicuspid aortic valves (P < 0.0001), age (P < 0.0001), diastolic blood pressure (P = 0.0008), body surface area (P = 0.015), and antihypertensive treatment (P = 0.005). Duration of oestrogen replacement therapy had an effect of borderline significance (P = 0.08). In this combined model karyotype 45,X (compared to any other karyotype), aortic valve function and elongated transverse aortic arch did not contribute. The modelling thus showed that aortic coarctation, bicuspid aortic valve, age, diastolic blood pressure, body surface area and antihypertensive medicines all influenced aortic diameter at least at one position. We subsequently studied the percentage change in aortic diameter that a given change in some of the studied variables led to, in order to gauge the effect sizes of these variables (Table 3). For example it could be appreciated that the presence of BAV increases the aortic diameter at the point of the aortic sinus with 10.39%, while receiving antihypertensive treatment reduces the diameter by 4.02% at the same measuring point.

**Table 2:** Initial modeling with relevant parameters thought to influence aortic diameter in Turner syndrome in a cohesive mathematical model

**Variable**	**CoA**	**Age**	**BAV**	**Antihypertensive treatment**	**Diastolic ABP**	**BSA**	**ERT duration**	**Karyotype**	**ETA**
**Position**									
Aortic sinus	0.04	0.0030	0.100	−0.041	−0.0006	0.17	0.0020	−0.06	0.0357
	(−0.04 - 0.12)	(0.0005 – 0.0056)	(0.038 – 0.160)	(−0.067 - -0.015)	(−0.0017 – 0.0005)	(−0.01 – 0.35)	(−0.0007 – 0.0046)	(−0.12 - -0.01)	(−0.0208 – 0.0923)
Sinotubular junction	−0.02	0.0031	0.129	−0.007	−0.0005	0.06	0.0017	−0.08	0.0165
	(−0.11 – 0.08)	(0.0003 – 0.0059)	(0.055 – 0.203)	(−0.034 – 0.018)	(−0.0017 – 0.0006)	(−0.14 – 0.25)	(−0.0013 – 0.0047)	(−0.14 - -0.01)	(−0.0515 – 0.0846)
Mid-ascending aorta	−0.06	0.0060	0.174	−0.010	0.0008	0.05	−0.0003	−0.06	0.0115
	(−0.16 – 0.04)	(0.0032 – 0.0089)	(0.097 – 0.252)	(−0.034 – 0.014)	(−0.0003 – 0.0018)	(−0.14 – 0.24)	(−0.0034 – 0.0027)	(−0.13 – 0.01)	(−0.0598 – 0.0828)
Distal ascending aorta	−0.03	0.0045	0.078	0.003	0.0008	0.10	−0.0022	−0.03	−0.00001
	(−0.11 – 0.05)	(0.0021 – 0.0068)	(0.015 – 0.141)	(−0.018 – 0.024)	(−0.0001 – 0.0017)	(−0.06 – 0.26)	(−0.0048 – 0.0003)	(−0.09 – 0.02)	(−0.0580 – 0.0579)
Proximal aortic arch	−0.07	0.0034	0.081	−0.012	0.0001	0.17	−0.0032	−0.05	−0.0108
	(−0.16 – 0.02)	(0.0009 – 0.0059)	(0.016 – 0.145)	(−0.037 – 0.014)	(−0.0011 – 0.0013)	(−0.001 – 0.35)	(−0.0058 – 0.0005)	(−0.11 – 0.01)	(−0.0702 – 0.0487)
Mid aortic arch	−0.06	0.0009	0.008	−0.008	0.0009	0.16	−0.0009	−0.02	−0.0456
	(−0.14 – 0.02)	(−0.0013 – 0.0032)	(−0.051 – 0.067)	(−0.029 – 0.013)	(−0.0001 – 0.0018)	(−0.002 – 0.31)	(−0.0033 – 0.0015)	(−0.07 – 0.04)	(−0.1000 – 0.0088)
Distal transverse aortic arch	−0.02	0.0026	0.024	0.006	0.0016	0.19	−0.0006	−0.03	−0.0241
	(−0.08 – 0.05)	(0.0006 – 0.0046)	(−0.025 – 0.073)	(−0.016 – 0.027)	(0.0007 – 0.0025)	(0.04 – 0.33)	(−0.0027 – 0.0016)	(−0.08 – 0.01)	(−0.0692 – 0.0210)
Proximal descending	0.21	0.0007	0.051	−0.014	0.0009	0.12	0.0001	−0.04	0.0037
	(0.13 – 0.29)	(−0.0016 – 0.0030)	(−0.009 – 0.110)	(−0.034 – 0.006)	(−0.0001 – 0.0017)	(−0.04 – 0.27)	(−0.0024 – 0.0025)	(−0.10 – 0.01)	(−0.0508 – 0.0583)
Distal descending aorta	0.14	0.0034	0.047	0.010	0.0014	0.20	−0.0008	−0.04	−0.0163
	(0.07 – 0.20)	(0.0016 – 0.0052)	(−0.0005 – 0.0094)	(−0.008 – 0.028)	(0.0006 – 0.0021)	(0.07 – 0.32)	(−0.0027 – 0.0012)	(−0.08 – 0.005)	(−0.0597 – 0.0270)
P value	**<0.0001**	**<0.0001**	**0.0004**	**0.005**	**0.0008**	**0.02**	**0.08**	**0.2**	**0.4**

The β estimates (95% confidence interval) are presented for each aortic position, and at the bottom of the table the p-value indicates whether the parameter influences the thoracic aorta at *any* point.

Abbreviations: *COARC* Aortic coactation, *BITRI* the presence of bicuspid aortic valve, *ABP* ambulatory blood pressure, *BSA* Body surface area, *ERT* oestrogen replacement therapy.

**Table 3:** **Percentage change in aortic diameter due to one unit increase in the variables influencing aortic diameter (based on the findings in Table **2**)**

**Variable**	**COARC**	**Age**	**BITRI**	**Antihypertensive treatment**	**Diastolic ABP**	**BSA**	**ERT duration**	**ETA**
**position**								
Aortic sinus	4.42	0.30	10.39	−4.02	−0.62	1.62	0.20	3.64
Sinotubular junction	−1,54	0.31	13.76	−0.74	−0.53	0.54	0.17	1.67
Mid-ascending aorta	−5.81	0.60	19.03	−0.99	0.78	0.52	−0.03	1.15
Distal ascending aorta	−3.10	0.45	8.12	0.31	0.84	0.96	−0.22	0.00
Proximal aortic arch	−6.73	0.34	8.39	−1.15	0.10	1.68	−0.31	−1.07
Mid aortic arch	−5.66	0.09	0.80	−0.77	0.86	1.49	−0.09	−4.45
Distal transverse aortic arch	−1.70	0.26	2.41	0.55	1.64	1.81	−0.06	−2.39
Proximal descending	23.40	0.07	5.19	−1.39	0.86	1.14	0.01	0.37
Distal descending aorta	14.43	0.34	4.77	1.00	1,37	1.94	−0.08	−1.62
**P value**	**<0.0001**	**<0.0001**	**0.0004**	**0.005**	**0.0008**	**0.02**	**0.08**	**0.4**

For BITRI, ETA and antihypertensive treatment we present the percentage change in diameter in the presence of these variables (in contrast to the normal condition of a tricuspid valve, no ETA and no antihypertensive treatment). For ERT duration we examined the additive effect of one additional year of treatment. For diastolic blood pressure we examined the percentage change in the aortic diameter due to a 10 mmHg increase and for BSA due to 10% increase. The p-value indicates whether the parameter influences the thoracic aorta at *any* point.

Abbreviations: *COARC* aortic coarctation, *BITRI* the presence of a bicuspid aortic valve, *ABP* ambulatory blood pressure, *BSA* Body surface area, *ERT* oestrogen replacement, *ETA* elongated transverse aortic arch.

We then proceeded to assess how these influencing variables determined aortic diameter by posing the question: is the nature of this influence uniform or does it vary from position to position through the thoracic aorta? These analyses showed that aortic diameter (at other positions), aortic coarctation, bicuspid aortic valve, age, and diastolic blood pressure had a position-dependent effect on aortic diameter, whereas body surface area exerted a position-independent effect on the thoracic aorta (Table 4). The aforementioned parameters had a stronger influence on the proximal ascending aorta than on the descending thoracic aorta. Even though the size of the interaction was position specific as described, the presence of bicuspid valves, aortic coarctation, raised blood pressure (and antihypertensive treatment), advanced age, and increased body surface area were all associated with increased aortic diameters (except around the classical site of aortic coarctation in individuals with such arch morphology). We also studied the effect of oestrogen replacement closer and this analysis showed that this treatment had a 'negative' effect on aortic diameter in the ascending aorta (position 1 and 2 – associated with a smaller aortic diameter), while it had a marginally positive influence on diameter in the remaining thoracic aorta.

**Table 4:** Integrating all nine aortic positions mapped by CMR in the forecasting model, producing interaction terms to describe the nature of the influence

**Variable**	**COARC**	**Age**	**BITRI**	**Antihypertensive treatment**	**Diastolic ABP**	**BSA**	**ERT duration**
Aortic sinus	0.06	0.002	0.11	−0.041	−0.0006	0.20	0.0025
	(−0.02 - 0.14)	(−0.0002 – 0005)	(0.05 – 0.17)	(−0.067 - -0.015)	(−0.0017 – 0.0005)	(0.02 – 0.38)	(−0.0002 - 0.0052)
Sinotubular junction	−0.002	0.003	0.14	−0.007	−0.0005	0.09	0.0023
	(−0.01 – 0.10)	(−0.0002 – 0.005)	(0.07 – 0.21)	(−0.032 – 0.019)	(−0.0016 – 0.0006)	(−0.11 – 0.28)	(−0.0007 - 0.0053)
Mid-ascending aorta	−0.05	0.006	0.18	−0.010	0.0008	0.07	0.00005
	(−0.15 – 0.05)	(0.0003 – 0.008)	(0.11 – 0.26)	(−0.034 – 0.014)	(−0.0002 – 0.0018)	(−0.12 – 0.26)	(−0.0030 - 0.0031)
Distal ascending aorta	−0.03	0.004	0.08	0.003	0.0009	0.11	−0.0021
	(−0.11 – 0.05)	(0.002 – 0.007)	(0.02 – 0.14)	(−0.018 – 0.023)	(0.00005 – 0.0017)	(−0.05 – 0.27)	(−0.0045 - 0.0004)
Proximal aortic arch	−0.07	0.003	0.08	−0.011	0.0001	0.19	−0.0028
	(−0.15 – 0.02)	(0.0007 – 0.006)	(0.02 – 0.14)	(−0.036 – 0.015)	(0.0010 – 0.0013)	(0.01 – 0.36)	(−0.0055 - -0.0002)
Mid aortic arch	−0.07	0.001	−0.003	−0.007	0.0009	0.15	−0.0010
	(−0.15 -0.01)	(−0.001 – 0.003)	(−0.06 – 0.05)	(−0.029 – 0.014)	(0.00004 – 0.0018)	(−0.01 – 0.30)	(−0.0034 - 0.0014)
Distal transverse aortic arch	−0.02	0.003	0.02	0.006	0.0017	0.20	−0.0003
	(−0.08 – 0.05)	(0.0006 – 0.004)	(−0.03 – 0.07)	(−0.015 – 0.028)	(0.0007 – 0.0026)	(0.06 – 0.34)	(−0.0024 - 0.0018)
Proximal descending	0.22	0.0004	0.05	−0.014	0.0009	0.13	0.0004
	(0.14 – 0.29)	(−0.002 – 0.003)	(−0.003 – 0.11)	(−0.034 – 0.006)	(−0.00002 – 0.0017)	(−0.02 – 0.29)	(−0.0020 - 0.0028)
Distal descending aorta	0.14	0.003	0.04	0.011	0.0014	0.21	−0.0005
	(0.08 – 0.20)	(0.002 – 0.005)	(−0.002 – 0.09)	(−0.007 – 0.029)	(0.0006 – 0.0022)	(0.08 – 0.34)	(−0.0025 - 0.0014)
**P value**	**<0.0001**	**<0.0001**	**0.0004**	**0.002**	**0.01**	**0.6**	**0.06**

Here, we present the interaction terms for parameters that showed an effect on the development in aortic diameter. The β estimates (95% confidence interval) are presented for all aortic positions, and at the bottom of the table p-values are presented. In cases where an interaction term shows an *insignificant* p-value this indicates that the parameter had a uniform effect on the development of the thoracic aortic diameter. A *significant* p-value indicates that a parameter influences the development of the thoracic aortic diameter in a variable fashion over time. For example, the presence of a bicuspid aortic valve influenced the entire thoracic aorta, but the effect was most pronounced in the ascending aorta.

Abbreviations: *COARC* aortic coarctation, *BITRI* the presence of a bicuspid aortic valve, *ABP* ambulatory blood pressure, *BSA* Body surface area, *ERT* oestrogen replacement.

Model checking included a normal probability plot of the standardised residuals and a scatter plot of the residuals against fitted values (Additional file 1: Figures S1 and S2), and both plots showed no departures from the basic assumptions. The plots were further subdivided with respect to time (3 groups) and to aortic position (9 groups), and these plots (not shown) confirmed the impression that the model fitted the data well.

### Modelling aortic diameter and growth

**Figure 2:**
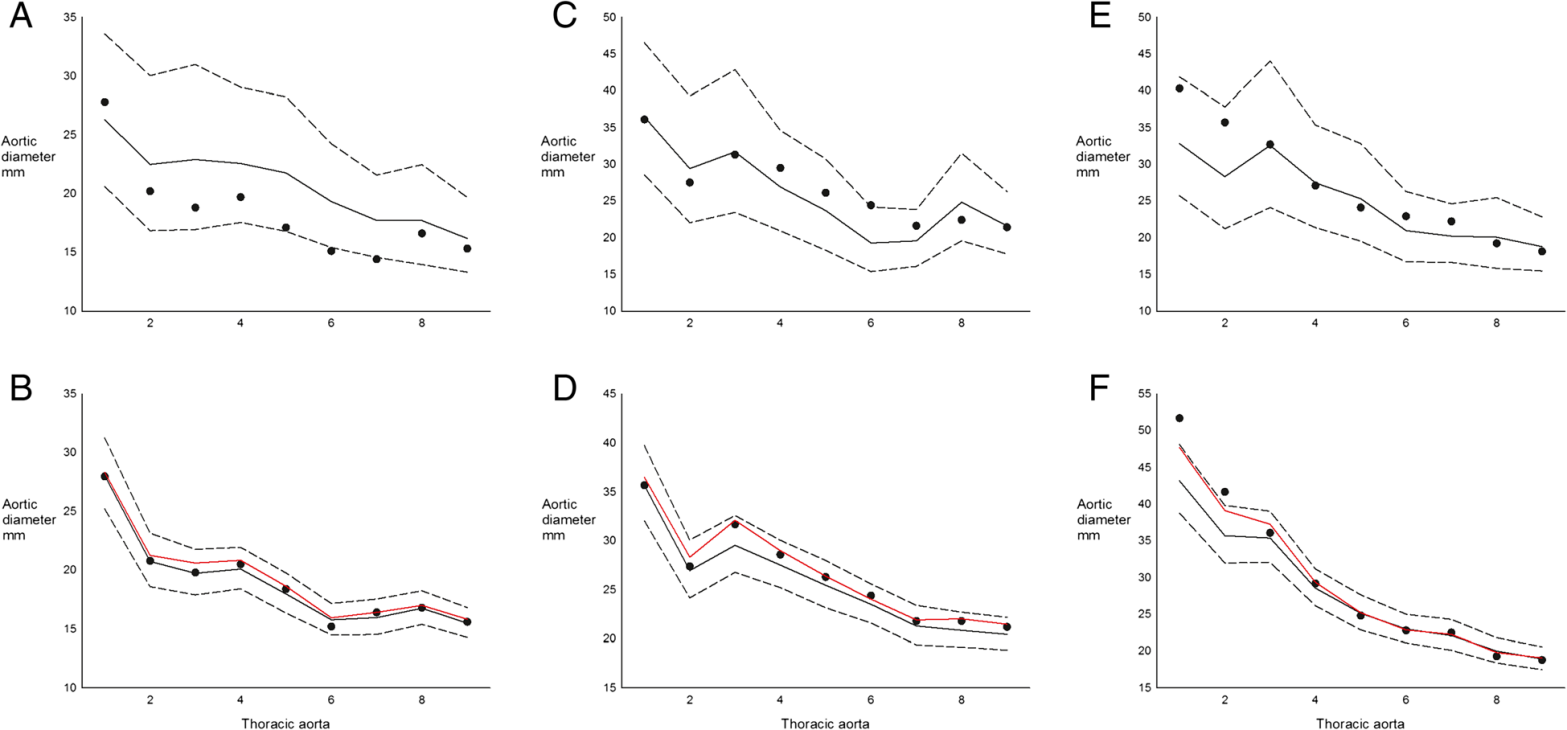
**Mathematical modelling of aortic diameter in Turner syndrome with varying burdens of risk factors for aortic complications, aiming to validate the predictive models against actual data collected in three real patients.** Aortic measurement position (nine: from aortic sinuses to descending thoracic aorta) is depicted on the x-axis. **A**) Perceived **low risk burden**: 26-year old, tricuspid aortic valve, no aortic coarctation, ambulatory blood pressure (ABP) 104/66 mmHg, body surface area (BSA) 1.46 m^2^, and karyotype 45,X (*dots*: actual measurement at baseline; *full black line*: prediction at baseline from modelling of the complete cohort (n = 102); *dotted lines*: 95% prediction limits). **B**) Same low-risk female (as in **A**) (*dots*: actual measurement at 4 years; *full black line*: 4 year prediction; *dotted lines*: 95% prediction limits; *full red line*: prediction at 8 years from baseline). **C**) Perceived **high risk burden**: 49-year old, bicuspid valves, aortic coarctation, hypertension (ABP 124/67 mmHg during antihypertensive treatment), BSA 1.60 m^2^, and karyotype 45,X/46,X,r(X) (*dotted line*: actual measurement at baseline; *full line*: prediction at baseline; *dotted lines*: 95% prediction limits). **D**) Same high-risk female (as in 2C) (*dots*: actual measurement at 4 years; *full black line*: 4 year prediction; *dotted lines*: 95% prediction limits; *full red line*: prediction at 8 years from baseline). **E**) Perceived **high risk burden**: 43-year old, bicuspid valves, no aortic coarctation, diagnosed hypertension (ABP 143/90 mmHg during antihypertensive treatment), BSA 1.47 m^2^, and karyotype 45,X (*dotted line*: actual measurement at baseline; *full line*: prediction at baseline; *dotted lines*: 95% prediction limits). **F**) Same high-risk female (as in **E**) (*dots*: actual measurements at 4 years; *full black line*: 4 year prediction; *dotted lines*: 95% prediction limits; *full red line*: 8 yeard prediction), and please note that ascending aortic measurements fell outside the prediction. Please also see http://www.biostat.au.dk/MERL/Aorta_Prediction_model.htm.

We designed a forecasting model, allowing prediction of changes in aortic diameter over time. This model was flexible, and allowed use in two versions. In one version, modelling can be performed without knowledge of prior aortic imaging and on the basis of other accessible data such as body surface area, blood pressure, and aortic valve status. In another version, baseline or even follow-up measurements of the aorta may be entered in addition to other risk factors. Not surprisingly, the accuracy of the modelling improved with the use of multiple aortic measurements. The prediction model also highlighted that it is possible to categorise females with TS into low and high risk for more or less rapid aortic dilation based on a number of risk parameters present, such as previous measurement of the aorta. In Figure 2A, 2C and 2E we present data from three females at different ends of the risk spectrum, running their data through the model. The figures demonstrate that risk factors additively predict progressive aortic dilation over time, especially in the ascending aorta, and that aortic growth primarily takes place in the ascending aorta closest to the aortic valves. Some females displayed growth according to prediction while others demonstrated rapid growth of the aorta exceeding the boundaries of the prediction limits of the model (Figure 2B, 2D and 2F), and we speculate that such individuals should be considered for more targeted medical or surgical intervention [9].

## Discussion

The entire thoracic aorta is at risk of dilation and dissection in TS [7,11]. The present study utilised a dynamic and integrative approach to both baseline CMR data and to the longest registered prospective characterisation of aortic diameter of the entire thoracic aorta in TS. The presented model provides a cohesive analysis of aortic disease by incorporating all parameters that have been identified to impact the thoracic aorta in TS with the goal to more completely estimate of the total risk burden. The mathematical modelling included every measurement performed in each individual at all study visits, creating a correlation matrix of more than 2,000 separately assessed aortic diameters.

Growth of the thoracic aorta was dynamic over time, and the presently acknowledged risk factors impacted diameters in different ways at different positions within the thoracic aorta. Risk factors such as age, bicuspid aortic valves, blood pressure, antihypertensive treatment, and aortic coarctation preferentially accelerated growth of the ascending aorta. Higher body surface area associated with larger aortic diameters, but in contrast to the former variables the interaction of body surface area was uniform across the thoracic aorta. Our findings emphasise that even though some risk factors may be congenital, there is a window of opportunity to prevent (or at least reduce) the age-related progressive aortic dilatation. Notably, hypertension is extremely frequent in TS, [18-20] and medical intervention may both reduce hypertensive wall shear stress and directly inhibit aortic wall disease [21,22] though drug trials are lacking in TS [1]. Hypertension was highly prevalent, markedly underdiagnosed and undertreated at baseline. Aortic diameter was higher in females who were being treated for hypertension, which is not due to a negative influence of antihypertensive drugs but rather the result of their underlying hypertensive disease. Hypertension is not the only modifiable risk factor. Careful handling of obesity and metabolic diseases that are common in TS may also improve aortic outcomes since aortic diameters are closely linked with body surface area [23]. As for the congenital risk factors, aortic valve morphology and aortic coarctation were both significant contributors whereas karyotype did not explain the variation in aortic diameter in our models. Previously, the 45,X karyotype has been established to associate with unfavourable aortic phenotypes in TS [10,24]. The lack of an association in our analyses is likely due to karyotype having an indirect influence via the associated traits such as bicuspid aortic valves and aortic coarctation.

Aortic growth rate is used as an additional risk factor for aortic dissection [9]. Here, the longest aortic follow-up in TS confirms the findings of previous studies [11,25], underpinning the aggressive nature of aortic disease in TS with growth rates that here ranged from 0.20 to 0.38 mm/year (and even higher rates were seen in a few patients in the ascending aorta). These growth rates are well beyond the general population and compares to other cohorts with aortic disease [26,27].

Our models infer that although the association between growth rates and age may seem linear, the nature of this relation is highly dependent on the position in the thoracic aorta. In clinical terms this means that accelerated growth may be present at certain positions along the aorta, whereas other positions undergo less pronounced change (and this picture may change over time and with interventions). The present model also highlights that a factor such as age is a principal player in the progression towards aortic dilation. Aortic dissection occurs four decades prematurely in TS and our findings reiterate that triaging for follow-up also should include the patients’ age [28].

The pervasive mapping of aortic phenotype with CMR over multiple visits enabled us to provide the first integrative model of the thoracic aorta in TS (Figure 2). Our models provide a ‘risk engine’ that predicts aortic diameter of a female with TS based on her actual risk factors. This may prove a valuable tool in the clinical assessment of aortic disease, when a female with TS is seen for the first time or when new risk factors are diagnosed in clinic as can be expected from the natural course of the syndrome [29]. By use of the model the clinician can compare any measured aortic size of a particular patient to the models reference values, and hereby get an understanding of the degree of enlargement of the aorta compared with similar patients. In addition, it is possible to ascertain a predicted impact of an occurrence such as onset of hypertension, which may aid in scheduling the most appropriate and timely follow-up or possibly guide interventions. Our models clearly show that the impact of different risk factors is far from trivial, and that all should be included in the planning of surveillance of the thoracic aorta in TS. Important for the external validity of this predictive model, the registration of aortic diameter was not only performed by gold standard imaging [3,9] but also carried out in a clinically applicable setting where risk modifications were adapted as cardiovascular risk factors emerged. This is in contrast to a controlled clinical setting that would not translate into a model of daily clinical practice.

It would have been optimal to link this model with aortic dissection and rupture, but no definitive aortic event occurred in this cohort despite several participants being excluded from follow-up due to cardiothoracic surgery and death of unknown cause. There is a lack of evidence-based guidelines for planning of monitoring and interventions for the dilated aorta in TS [3,9,30]. A critical cut-off level for aortic diameter remains to be defined. In light of the shortage of evidence, we had to take a pragmatic approach in two females with gross dilation of their ascending aorta. At baseline, a 24-year old female with a mid-ascending aortic diameter of 48 mm and bicuspid aortic valve had aortic surgery. Unfortunately, she died immediately after surgery due to complications relating to an aberrant origin of her left anterior descending coronary artery (origin from the distal ascending aorta). At the end of the study, a 43-year old female (Figure 2E), who presented with a bicuspid aortic valve and progressed in her aortic sinus diameter from 40 to 52 mm over 5 years, had the same surgical procedure performed with a good result. These cases illustrate the paradigm associated with gathering outcome-based evidence for aortic diameter and dissection in TS, as it is ethically difficult to carry out truly observational studies without intervening. It also needs to be remembered that even though the risk of aortic dissection may be 100-fold increased, this event is not a common clinical occurrence, since it will only affect 1-2% of TS over their entire lifetime. We will continue to explore outcomes in this cohort, and hopefully provide more insight into the relation between aortic diameter and aortic events in TS.

### Future perspectives

Our models are the first of their kind for disease in the thoracic aorta. They lend promise of becoming a valuable clinical tool that should optimally be validated in other large cohorts of TS (including children and adolescents). Further modelling might even include similar ‘risk engines’ that predict aortic diameter using CMR on a population level, where TS might be one of many risk factors entered into the models alongside age, bicuspid aortic valve, aortic coarctation, body surface area, Marfan syndrome or other thoracic aortic dilation syndromes. Improving outcomes in aortic disease is pivotal, and our work adds to other important areas of focus in the improvement of prognosis in aortic disease [9]. Linearity has hither to been assumed when it comes to modelling aortic growth and when using serial studies for risk assessment, [11,26,27] but according to our data this may be a somewhat crude assumption. This complicates assessment of change between any two studies (and the clinical implications hereof) because a change may vary highly with time in a non-linear fashion. There is, as proposed here, a need for inclusion of more factors than time and diameter, and it will in the future be essential to clarify how different parts of the aorta grow differentially over different time periods and in the presence of different combinations of risk factors.

### Study limitations

The model treated all participants lost to follow-up (n = 20) as non-informative (or ‘missing at random’ in the mathematical terminology). Of these, some females (n = 13) were lost for non-health related reasons and no events occurred here; the assumption that this loss to follow-up is non-informative seems reasonable. For the remaining participants (n = 7) that were lost to follow-up, they either were or could have been lost for reasons associated with aortic diameter. Hence, the assumption that they were ‘missing at random’ may be violated. Such an association could also create a selection problem in the data similar to the healthy worker effect and hence introduce bias in the estimates. However, the number of exits is too small for a more extensive mathematical evaluation.

Some limitations in the autoregressive temporal relationship are imposed by existing software (SAS PROC MIXED) and improvements could be obtained by addressing two aspects: (i) multivariate (spatial) data can only be fitted as an auto-regression with constant time interval between the observations, approximately 2 years for this study, and hence the Kalman filter based on our estimates can only be applied in similar situations; and (ii) the autoregressive model with only a single parameter common for all aorta positions is very intuitive but could be too simplistic. Such improvements would, however, require much more specialised software.

## Conclusion

The robust mathematical model shows the importance of several risk factors for dilation of the thoracic aorta in TS. This cohesive model for prediction of aortic diameter, which included all aortic measurements for several time points at the same time, could serve to identify patients with rapid growth of aortic diameter, to aid clinical decision-making based on CMR studies, and may also prove useful in future imaging studies.

## Competing interests

The authors declare that they have no competing interests.

## Authors’ contributions

CHG conceived the study, with contributions to the design, coordination and conduction from KHM, NHA and ME. CMR was performed and handled by KHM. Echocardiography was performed by KHM and NHA. CHG, ME and KHM performed the statistical analyses and drafted the manuscript with substantial contributions from all other authors. All authors read and approved the final manuscript.

## Supplementary Material

Additional file 1 Principles of the mathematical modelling used to forecast aortic diameter in Turner syndrome from prospective CMR data collected over nearly 5 years in a prospective cohort study. Click here for file
