# Supplementary material for: Prediction of aortic dilation in Turner syndrome - enhancing the use of serial cardiovascular magnetic resonance
Source: J Cardiovasc Magn Reson. 2013 Jun 6;15(1):47. doi: 10.1186/1532-429X-15-47 (PMC3702474; doi:10.1186/1532-429X-15-47)
Supplement: Additional file 1 — Principles of the mathematical modelling used to forecast aortic diameter in Turner syndrome from prospective CMR data collected over nearly 5 years in a prospective cohort study. [file 1532-429X-15-47-S1.docx]

**ONLINE SUPPLEMENTAL FILE**

***The mathematical model***

The multivariate (*N* positions) multiple (*p* regressors) regression model is composed of a measurement equation (1) and a transition equation for the “system noise” (2):

$$\begin{matrix} (1) \mathbf{y}_{t}=\mathbf{Bx}_{t}+\mathbf{w}_{t}+\mathbf{e}_{t}, \mathbf{e}_{t}\sim\mathbf{N}_{N}(\mathbf{0},\mathbf{R}) \mathbf{R}=diag(\sigma_{1}^{2},...,\sigma_{N}^{2}) \\ (2) \mathbf{w}_{t}=\rho\mathbf{w}_{t-1}+\mathbf{v}_{t}, \mathbf{v}_{t}\sim\mathbf{N}_{N}(\mathbf{0},\mathbf{\Sigma}) \end{matrix}$$

where $\mathbf{y}_{t}$ is the *N* x 1 observed response vector at time *t*, **B** is an unknown N x *p* regression coefficient matrix, $\mathbf{x}_{t}$ is the *p* x 1 vector of observed regressors at time *t*, $\mathbf{w}_{t}$ is a *p* x 1 vector of random “system noise”, $\mathbf{e}_{t}$ is a *p* x 1 vector of independent measurement errors, *ρ* is an unknown autoregressive parameter and $\mathbf{v}_{t}$ is a *p* x 1 vector of independent system errors.

The model can be fitted with SAS PROC MIXED using a Kronecker product structure on the covariance matrix on the observations (UN@AR(1) with the ‘local’ option, please see references for further detail. [^1^](#_ENREF_1)

A more general model can be obtained by replacing the autoregressive parameter *ρ* with a possible time-dependent transition matrix $\mathbf{\Phi}_{t}$, but such a model requires more specialized software.

***The prediction model***

Baseline predictions without any prior measurements of the aorta diameter are obtained from the prediction equation using the estimated coefficient matrix ($\hat{\mathbf{B}}$) and the available information on the regressor ($\mathbf{x}_{t}$) as $\hat{\mathbf{y}}_{t}=\hat{\mathbf{B}}\mathbf{x}_{t}$. The prediction limits for each position are obtained from the diagonal of the estimated covariance matrix .

***The forecasting model***

If *t* measurements are available ($\mathbf{y}_{1},\mathbf{y}_{2},...,\mathbf{y}_{t}$) and the regressor at time *t*+1 is known (or prespecified) the optimal forecast of $\mathbf{y}_{t+1}$using all prior information can be obtained with the Kalman filter using the prediction and updating equations on the system noise ($\mathbf{w}_{t}$), please see references for further detail. ^2, 3^

Prediction equations:

Updating equations:

$$\begin{matrix} \hat{\mathbf{w}}_{t| t}=\hat{\mathbf{w}}_{t| t-1}+\mathbf{P}_{t| t-1}\mathbf{F}_{t}^{-1}(\mathbf{y}_{t}-\mathbf{Bx}_{t}-\mathbf{w}_{t| t-1}) \\ \mathbf{P}_{t}=\mathbf{P}_{t| t-1}-\mathbf{P}_{t| t-1}\mathbf{F}_{t}^{-1}\mathbf{P}_{t| t-1} \\ \mathbf{F}_{t}=\mathbf{P}_{t| t-1}+\mathbf{R} \end{matrix}$$

Unknown parameters are replaced by estimated values. The prediction limits for each position are obtained from the diagonal elements of

$\mathrm{cov}(\mathbf{y}_{t+1| t})=\mathbf{F}_{t+1}=\mathbf{P}_{t+1| t}+\mathbf{R}$.

***Prediction limits and prediction sets***

The prediction limits calculated and shown in figures 2A-2F are “one-dimensional” or marginal limits in the sense that they are calculated for each position without taking into account the high positive correlation between all nine positions. This problem is similar to the problems with generating multivariate confidence limits (and multiple testing procedures) and there is no single solution to calculate multivariate prediction limits, i.e. a 9-dimensional prediction set. One choice is a multivariate ellipsoid based on the 9-dimensional multivariate normal distribution, but it is not simple to illustrate graphically. If all parameters were known we could calculate a $1-\alpha$ prediction set $(PS_{1-\alpha})$ using (please see Kalman filter equations above) and hence

Thus $PS_{1-\alpha}=\left\{\mathbf{y}_{P}|X^{2}(\mathbf{y}_{P})<\chi_{1-a}^{2}(DF=9)\right\}$ would be a $1-\alpha$ prediction set.

Applying this idea to the female shown in Figure 2E/2F, we can calculate the probability of an observation at least as extreme as the observed at *t*+1 years by using the Kalman filter to obtain and $\mathbf{F}_{t+1}=\mathbf{P}_{t+1| t}+\mathbf{R}$. For the female shown in Figure 2E/2F the 2 and 4 years probabilities are 0.53 (data not shown) and 0.015. We note, that these calculations strictly speaking are not valid, since the parameters are unkown and this female has contributed to the estimated values of the parameters.

***Checking the mathematical model***

**Supplemental Figure A.**

Q-Q plot of all residuals with all available data in the model. The plot shows a good agreement with a standard normal distribution.

**Supplemental Figure B.**Scatter plot of residuals versus predicted values.

**REFERENCES**

1. Thiébaut R, Jacqmin-Gadda H, Chêne G, Leport C, Commenges D. Bivariate linear mixed models

using SAS proc MIXED. Computer Methods and Programs in Biomedicine; 2002; 69;249-256.

2. Cary NC. SAS/IML 9.1 users guide. SAS Institute Inc; 2004

3. Harvey AC. Forecasting, structural time series models and the kalman filter. Cambridge University Press; 1989
